# Increasing fetal ovine number per gestation alters fetal plasma clinical chemistry values

**DOI:** 10.14814/phy2.12905

**Published:** 2016-08-26

**Authors:** Micaela Zywicki, Sharon E. Blohowiak, Ronald R. Magness, Jeffrey L. Segar, Pamela J. Kling

**Affiliations:** ^1^ Departments of PediatricsUniversity of Wisconsin‐Madison School of Medicine and Public HealthMadisonWIUSA; ^2^ Obstetrics and Gynecology Perinatal Research LaboratoriesUniversity of Wisconsin‐Madison School of Medicine and Public HealthMadisonWIUSA; ^3^ Department of PediatricsUniversity of Iowa Children's HospitalIowaIAUSA

**Keywords:** Fetal, nutrition, ovine, placenta

## Abstract

Intrauterine growth restriction (IUGR) is interconnected with developmental programming of lifelong pathophysiology. IUGR is seen in human multifetal pregnancies, with stepwise rises in fetal numbers interfering with placental nutrient delivery. It remains unknown whether fetal blood analyses would reflect fetal nutrition, liver, and excretory function in the last trimester of human or ovine IUGR. In an ovine model, we *hypothesized* that fetal plasma biochemical values would reflect progressive placental, fetal liver, and fetal kidney dysfunction as the number of fetuses per gestation rose. To determine fetal plasma biochemical values in singleton, twin, triplet, and quadruplet/quintuplet ovine gestation, we investigated morphometric measures and comprehensive metabolic panels with nutritional measures, liver enzymes, and placental and fetal kidney excretory measures at gestational day (GD) 130 (90% gestation). As anticipated, placental dysfunction was supported by a stepwise fall in fetal weight, fetal plasma glucose, and triglyceride levels as fetal number per ewe rose. Fetal glucose and triglycerides were directly related to fetal weight. Plasma creatinine, reflecting fetal renal excretory function, and plasma cholesterol, reflecting placental excretory function, were inversely correlated with fetal weight. Progressive biochemical disturbances and growth restriction accompanied the rise in fetal number. Understanding the compensatory and adaptive responses of growth‐restricted fetuses at the biochemical level may help explain how metabolic pathways in growth restriction can be predetermined at birth. This physiological understanding is important for clinical care and generating interventional strategies to prevent altered developmental programming in multifetal gestation.

## Introduction

More than 3% of human pregnancies carry multiple fetuses (Martin et al. 2010). When uterine space for implantation is limited, intrauterine growth restriction (IUGR) can develop, often resulting in perinatal morbidity and even fetal mortality. Defining the critical point of deteriorating fetal function in IUGR has been of much clinical interest (Cox et al. 1988). Additional research is needed to understand biomarkers of fetal health deterioration in IUGR. This is especially true under the common conditions of uterine space restriction (USR) from either uterine anomalies or multifetal gestation pregnancies.

Ovine gestational models have been used to study placental insufficiency and other causes of IUGR, with maternal–fetal blood sampling allowing better understanding of oxygen and nutrient transfer by the placenta (Barry and Anthony 2008). The vascular structures of both human and ovine placenta share many similarities, with an additional benefit of discrete cotyledons in the ovine placenta allowing precise quantification of placental attachment sites (Meyer et al. 2010). However, limited biochemical information is available for the study of USR‐induced growth restriction (Meyer et al. 2010; Meyer‐Gesch et al. 2013).

We previously reported that with restricted uterine space for placental implantation, changes in placental efficiency and deficits in placental nutrient delivery cause compensatory adaptations to promote a viable, albeit compromised fetus (Meyer et al. 2010; Meyer‐Gesch et al. 2013); however, these alterations may become maladaptive later in life, altering metabolic pathways and promoting adult diseases, including hypertension and diabetes as described in the Barker hypothesis (Anthony et al. 2003; de Boo and Harding 2006). In addition, studies found that ewes carrying multiple fetuses exhibited decreased birth weight and survival of the lambs (Gootwine et al. 2007). By examining plasma biochemical markers of ovine placental–fetal nutrition, fetal liver enzymes, and placental–fetal excretory function associated with growth restriction, we could identify fetal biomarkers of maladaptive compensation.

Understanding these fetal blood biochemical biomarkers may also explain how fetal dysfunction in growth restriction impacts the developmental programming of adult disease and ultimately to aid understanding of potential therapeutic strategies. Although data were also obtained from a cohort at gestational day (GD) 120 and are for reference in the supplementary materials, relationships between fetal number and clinical chemistries at GD130 would better inform underlying physiology because growth arrest became apparent between GD120 and GD130. In an ovine model, it was *hypothesized* that fetal plasma biochemical values would reflect progressive placental, fetal liver, and fetal kidney dysfunction as the number of fetuses per gestation rose. The *aims* of this study are to provide fetal plasma biochemical reference values for future research in ovine multifetal gestation and utilize these biochemical values in examining relationships between uterine–placental space restriction and fetal growth.

## Methods

### Animals

Mixed Western breed ewes previously selected for the *Polypay* and *Booroola* genes were obtained from the University of Wisconsin‐Madison farm facility, group‐housed, and fed identical diets that were a mixture of hay and corn silage that met daily NRC feed requirements of pregnant sheep (Meyer et al. 2010; Meyer‐Gesch et al. 2013). The University of Wisconsin‐Madison Research Animal Care and Use Committee of the School of Medicine and Public Health and the College of Agriculture and Life Sciences approved the protocols. To accurately determine gestational age, a synchronization protocol was performed using an intravaginal controlled internal drug release (Progesterone CIDR; 0.3 g; Pfizer, Aukland, New Zealand) for 10–14 days, followed by intramuscular prostaglandin F_2_ *α* (15 mg; Pfizer) and equine chorionic gonadotropin (500 IU; EMD Biosciences, San Diego, CA) injections before breeding (Meyer‐Gesch et al. 2013; Sun et al. 2013). Pregnancy was confirmed using ultrasound by GD60.

### Surgical procedures

The study's intent was to investigate the impact of multifetal gestation without any previous procedural manipulation to decrease uterine space. Nonsurvival surgery was performed at GD120 or GD130 (±4 days), with term at GD145. GD120, or 80% gestation, was selected because previous data showed only subtle impact of uterine space restriction with more than one fetus per uterine horn (Meyer et al. 2010). GD130, or 90% gestation, was selected because asymmetric IUGR became apparent with more than one fetus per horn (Meyer et al. 2010; Meyer‐Gesch et al. 2013). After administering sodium pentobarbital and ketamine anesthesia to the pregnant ewe, a uterine incision was performed and simultaneous umbilical artery and vein blood sampling (12 mL) with a 20G needle was performed from each fetus and the collected blood immediately placed in heparinized tubes that were kept on ice. Fetal blood samples were collected from anesthetized ewes without supplemental oxygen, at an estimated time of 25 min from the phenobarbital administration and 15–20 min after the ewe was placed supine on the table. Mean maternal artery pO_2_ was 55.5 and mean fetal umbilical vein pO_2_ was 22.4 mmHg. After blood sampling, the ewe and fetuses were euthanized and fetal organ morphometry was obtained.

Placental and body growth was assessed by placentome count, fetal body weight (kg), kidney weight (g), liver weight (g), and brain weight (g) (Meyer et al. 2010). Plasma chemistries were assessed after centrifugation of heparinized blood for 10 minutes at 3600 X g at 4°C. A comprehensive metabolic panel was measured within 5 h in the Clinical Chemistry Laboratory at Meriter Laboratories using the Cobas Integra 800 (Roche Pharmaceuticals, Basel, Switzerland). Plasma macronutrients and minerals included glucose (mg/dL), triglycerides (mg/dL), albumin (g/dL), and total protein (g/dL), as well as Alkaline (Alk) Phosphatase (U/L) representing calcium metabolism. Plasma liver enzymes measured include aspartase transaminase (AST) (U/L), gamma‐glutamyl transpeptidase (GGT) (U/L), alanine transaminase (ALT) (U/L), and lactate dehydrogenase (LDH) (U/L). Tests of placental and fetal excretory function included cholesterol (mg/dL), creatinine (mg/dL), BUN (mg/dL), and sodium (mmol/L). Additional methods included plasma iron (*μ*g/dL) (Cobas Integra), iron binding capacity (*μ*g/dL), and transferrin saturation (%) from Pointe Scientific, and hematocrit % (pocH‐100i, Sysmex, Mundelein, IL), see Tables S1 and S2.

Normality of distribution was assessed. Nonparametric data were natural log (ln)‐converted to normalize the distribution. Analysis of Variance (ANOVA) with Fisher's post hoc analyses compared fetal organ weight or chemistry values by fetal lamb number per ewe. Simple regression analyses were performed by comparing fetal organ or chemistry values by fetal weight and the highest order relationship models are reported. Data S1 show 95% confidence intervals for parameters. Also included are qualitative directional comparisons of fetal lamb values with human fetal growth restriction at the comparable analogous time points in gestation. “Placental dysfunction” in multifetal gestation was defined as the point when inadequate space for placentomal attachment sufficiently disrupts fetal nutrient delivery resulting in fetal growth restriction, altered nutritional or metabolic parameters, or placental clearance to impact fetal biochemical indices. Although it is typically a label used for postnatal live born lambs, “small for gestational age (SGA)” was defined as <10% percentile for our population, fetal singleton carcass weight within that gestational age group.

## Results

### Placental and body growth

**Figure 1:**
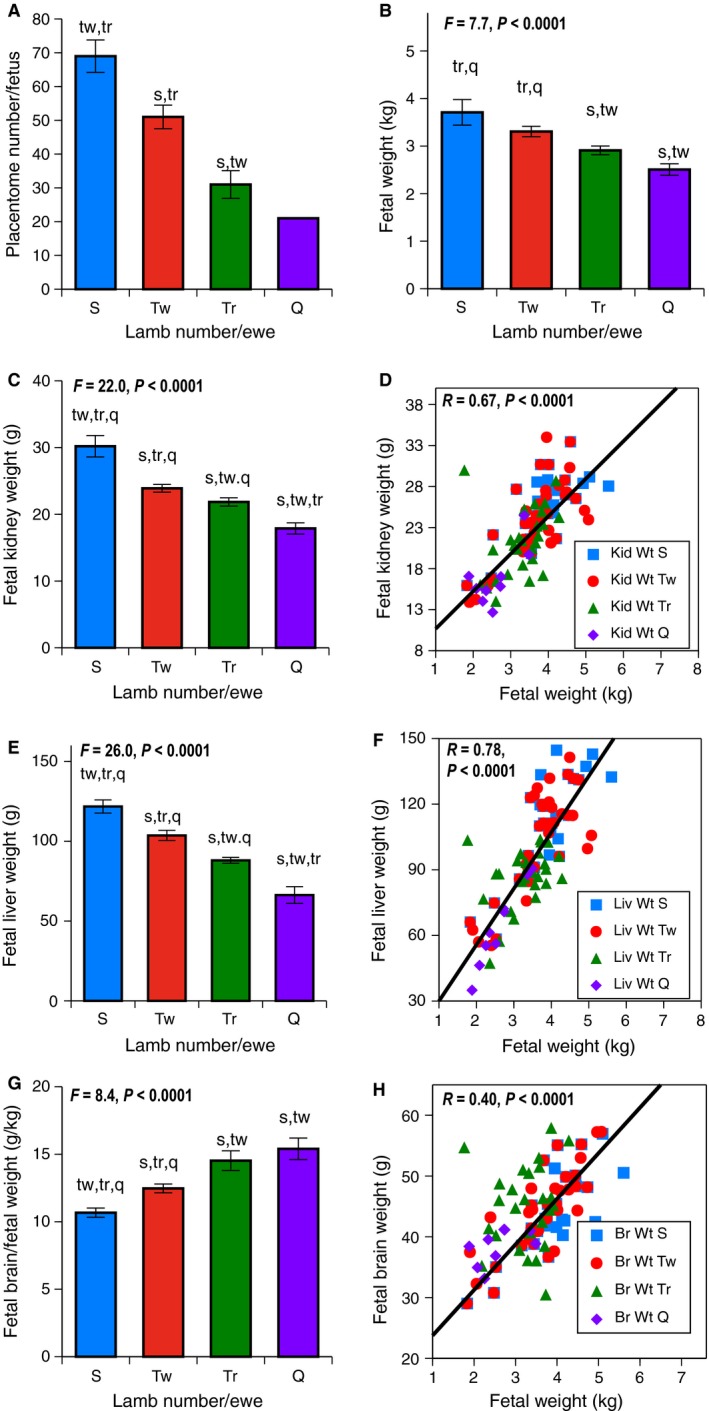
Growth Parameters at GD130 on the vertical axis were examined based on fetal number per ewe with S (singletons blue), Tw (twins red), Tr (triplets green), and Q (quadruplets/quintuplets purple) on horizontal axis. Error bars represent Means ± SEM. Lower case letters indicate post hoc differences (s from singletons, tw from twins, tr from triplet, and q from quadruplets/quintuplets). Regression lines follow the same color scheme; S in squares, Tw in circles, Tr in triangles, and Q in diamonds. (A) Placentomes; as fetal number per ewe increased, a stepwise fall in placentome number was seen, *P *< 0.001. (B) Fetal Weight (kg); as fetal number per ewe increased, a stepwise fall in fetal weight was seen, *P *< 0.0001. Samples sizes were 14 singletons, 45 twins, 34 triplets, and 8 quadruplets+. (C) Fetal Kidney Weight (g); as fetal number per ewe increased, a stepwise fall in fetal kidney weight was seen, *P *< 0.0001. Sample sizes were 18 singletons, 58 twins, 42 triplets, and 14 quadruplets+. D. Fetal Kidney Weight (g) and Fetal Weight (kg). Kidney weight was directly related to fetal weight, *P *< 0.0001. (E) Fetal Liver Weight (g); as fetal number per ewe increased, a stepwise fall in fetal liver weight was seen, *P *< 0.0001. Sample sizes were 16 singletons, 56 twins, 42 triplets, and 14 quadruplets+. (F) Fetal Liver Weight (g) and Fetal Weight (kg). Liver weight was directly related to fetal weight, *P *< 0.0001. (G) Fetal Brain Weight (g) Relative to Fetal Weight (kg); Fetal brain weight was relatively spared, with stepwise increase observed when expressed relative to fetal weight, *P *< 0.0001. Sample sizes were 16 singletons, 52 twins, 40 triplets, and 8 quadruplets+. (H) Fetal Brain Weight (g) and Fetal Weight (kg). Brain weight was directly related with fetal weight, *P *< 0.0001.

The multiparous ewes ranged from 2.1 years to 9.79 at delivery, although ewe age was not recorded in a few instances. Mean age of the ewes delivering singletons was 4.54 years, twins 4.48 years, triplets 4.59 years, and quadruplets+ 4.92 years, with no differences in ewe age when compared by fetal number. As the onset of growth restriction fell between GD120–GD130 (Meyer et al. 2010; Meyer‐Gesch et al. 2013), we examined and found that the mean gestational age did not differ between the singleton, twin, and triplet and the quadruplet and quintuplet groups. The 10th percentile for GD130 singleton fetuses in our flock is 2.626 kg and defines SGA. It was found that 10.8% of singletons were SGA, 26.8% of twins, 34.7% of triplets, and 61.5% of quadruplet and quintuplet group were SGA. The figures display data from GD130 data because previously (Meyer et al. 2010; Meyer‐Gesch et al. 2013), in a mixed group that included both surgically reduced uterine space and naturally decreased due to multifetal gestation, growth and clinical chemistry values showed more variability based on fetal number per ewe at GD130. Reference values grouped by natural singletons, twins, triplets, or quadruplet/quintuplets at both GD120 and GD130 are located in Data S1. The number of discrete placentome attachment sites fell as number of fetuses per ewe increased (*P *<* *0.0001), supporting a loss in maternal–fetal connections, (Fig. 1A). At GD130, fetal weight (kg) exhibited a stepwise fall as fetal number per ewe increased, *P *<* *0.0001, (Fig. 1B). Fetal kidney weight at GD130 fell in a graded fashion as fetal number per ewe rose, *P *<* *0.0001 (Fig. 1C). Because fetal weight was so strongly related to fetal number, we utilized fetal weight as the surrogate for space restriction caused by greater fetal number, a direct linear relationship was found between fetal weight and fetal kidney weight at GD130 (*P *<* *0.0001), (Fig. 1D). Kidney weight (g) expressed relative to fetal weight (kg) was unchanged as fetal number per ewe rose. A stepwise fall in fetal liver weight at GD130 was observed as fetal number per ewe rose (*P *<* *0.0001), (Fig. 1E), and was directly related to fetal weight at GD130 (*P *<* *0.0001), (Fig. 1F). Liver weight (g) expressed relative to fetal weight (kg) did not differ as fetal number per ewe rose, until 4 when it was lower than fetal weight (*P *<* *0.01). At GD130, brain weight (g) did not differ until fetal number reached 4 at which point brain weight fell (*P *<* *0.02), but fetal brain weight expressed proportionate to fetal weight (g/kg) exhibited a stepwise rise as fetal number per ewe rose (*P *<* *0.0001), (Fig. 1G), and was directly related to fetal weight (*P *<* *0.0001), (Fig. 1H).

Male fetuses weighed more than females (kg) in each fetal number grouping, *P *<* *0.0001, but BMI did not differ by sex in these groupings. Liver and kidneys organ weights (g) expressed relative to fetal weight (kg) did not differ by sex, although these organ weights (g) in twins and triplets were lower in females (*P *<* *0.05). Brain weights (g) did not differ by sex, although brain weights (g) expressed relative to fetal weight (kg) in twins and triplets were lower in males (*P *<* *0.03).

### Observations on sample source sex differences

In clinical chemistry values with paired data, there were no measurable differences between fetal vein and fetal artery. Comparing clinical chemistry values from singletons, twins, triplets, and quads/quints, the only difference by sex was glucose values lower in males when fetal number was at least 4, compared to females in that group.

### Macronutrients and minerals

**Figure 2:**
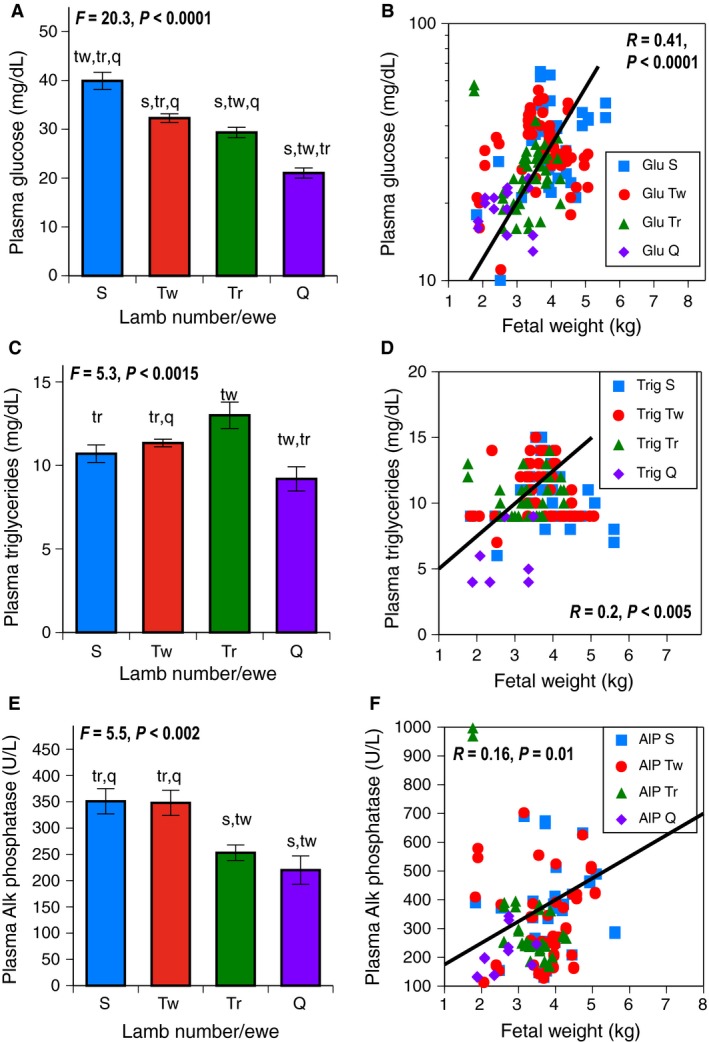
Macronutrients and Minerals at GD130 on the vertical axis were examined based on fetal number per ewe with S (singletons blue), Tw (twins red), Tr (triplets green), and Q (quadruplets/quintuplets purple) on horizontal axis. Error bars represent Means ± SEM. Lower case letters indicate post hoc differences (s from singletons, tw from twins, tr from triplet, q from quadruplets/quintuplets). Regression lines follow the same color scheme, S in squares, Tw in circles, Tr in triangles, and Q in diamonds. (A) Fetal Plasma Glucose levels (mg/dL); as fetal number per ewe increased, a stepwise fall in glucose was seen, *P *< 0.0001. Sample sizes were 18 singletons, 58 twins, 42 triplets, and 8 quadruplets+. (B) Plasma Glucose Levels (mg/dL) and Fetal Weight (Kg). Glucose was directly related to fetal weight, *P *< 0.0001. (C) Fetal Plasma Triglyceride levels (mg/dL); triglyceride differed, with lowest values in quads/quints, *P* < 0.0015. Sample sizes were 18 singletons, 58 twins, 40 triplets, and 8 quadruplets+. (D) Fetal Plasma Triglyceride levels (mg/dL) and Fetal Weight (kg). Plasma triglyceride was directly proportionate to fetal weight, *P *< 0.005. (E) Fetal Plasma Alkaline (Alk) Phosphatase levels (U/L); Alk Phosphatase was lower in triplets and quadruplets/quintuplets than other groups, *P *< 0.002. Sample sizes were 18 singletons, 58 twins, 42 triplets, and 8 quadruplets+. (F) Fetal Alk Phosphatase levels (U/L) and Fetal weight (kg). Alk Phosphatase was directly proportionate to fetal weight, *P *< 0.01.

At GD130, fetal plasma glucose levels fell in a stepwise fashion as total lambs per ewe increased (*P *<* *0.0001), (Fig. 2A). Fetal plasma glucose levels were directly related to fetal weight (*P *<* *0.0001), (Fig. 2B). Fetal plasma triglycerides levels were lower in the quadruplets/quintuplets than twins or triplets (*P *<* *0.015), (Fig. 2C) and were directly related to fetal weight (*P *<* *0.005), (Fig. 2D). Fetal plasma Alk Phosphatase levels, a biomarker of bone calcium metabolism, were lower in triplets and quadruplets/quintuplets (*P *<* *0.002), (Fig. 2E*),* and were directly related to fetal weight (*P *<* *0.01), (Fig. 2F). Additional measures of fetal iron nutrition are contained in the Data S1.

### Liver enzyme tests

**Figure 3:**
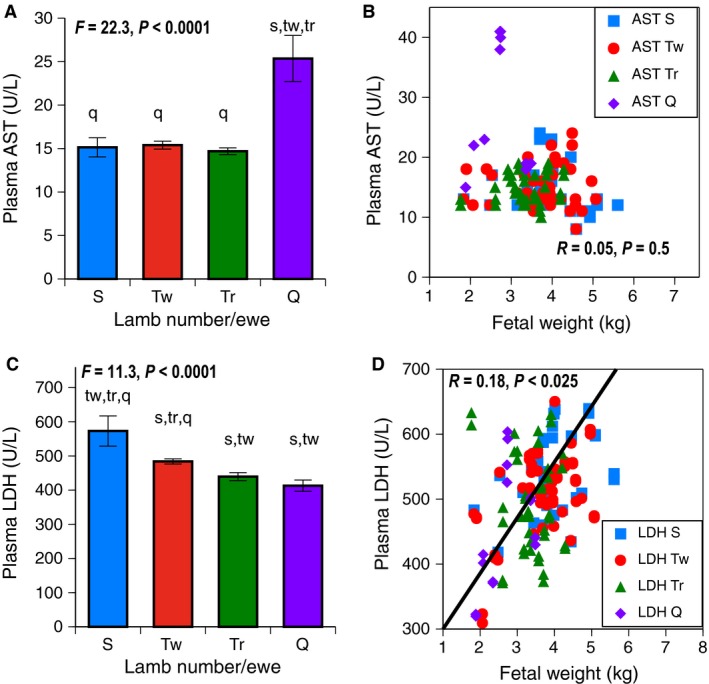
Liver Enzymes at GD130 on the vertical axis were examined based on fetal number per ewe with S (singletons in blue), Tw (twins red), Tr (triplets green), and Q (quadruplets/quintuplets purple) shown on the horizontal axis. Error bars represent Means ± SEM. Letters indicate post hoc differences from other groups (s from singletons, tw from twins, tr from triplets, q from quadruplets/quintuplets). Regression lines follow the same color scheme; S in squares, Tw in circles, Tr in triangles, and Q in diamonds. (A) Fetal Plasma AST levels (U/L); AST was increased in quadruplets/quintuplets, *P *< 0.0001. Sample sizes were 14 singletons, 56 twins, 42 triplets, and 14 quadruplets+. (B) Fetal Plasma AST levels (U/L) and Fetal Weight (kg). AST was not related to fetal weight. (C) Fetal Plasma LDH levels (U/L); LDH was lower in triplets and quadruplets/quintuplets, *P *< 0.0001. Sample sizes were 14 singletons, 56 twins, 36 triplets, and 14 quadruplets+. (D) Fetal LDH levels (U/L) and Fetal weight (kg). LDH was directly related to fetal weight (kg), *P *< 0.025.

At GD130, fetal plasma AST levels rose in the quadruplet/quintuplet group (*P *<* *0.0001), (Fig. 3A), but were unrelated to fetal weight, (Fig. 3B). Fetal plasma LDH levels fell as fetal number rose (*P *<* *0.0001), (Fig. 3C), and were also directly related to fetal weight (*P *<* *0.025), (Fig. 3D).

### Placental and fetal renal excretory function tests

**Figure 4:**
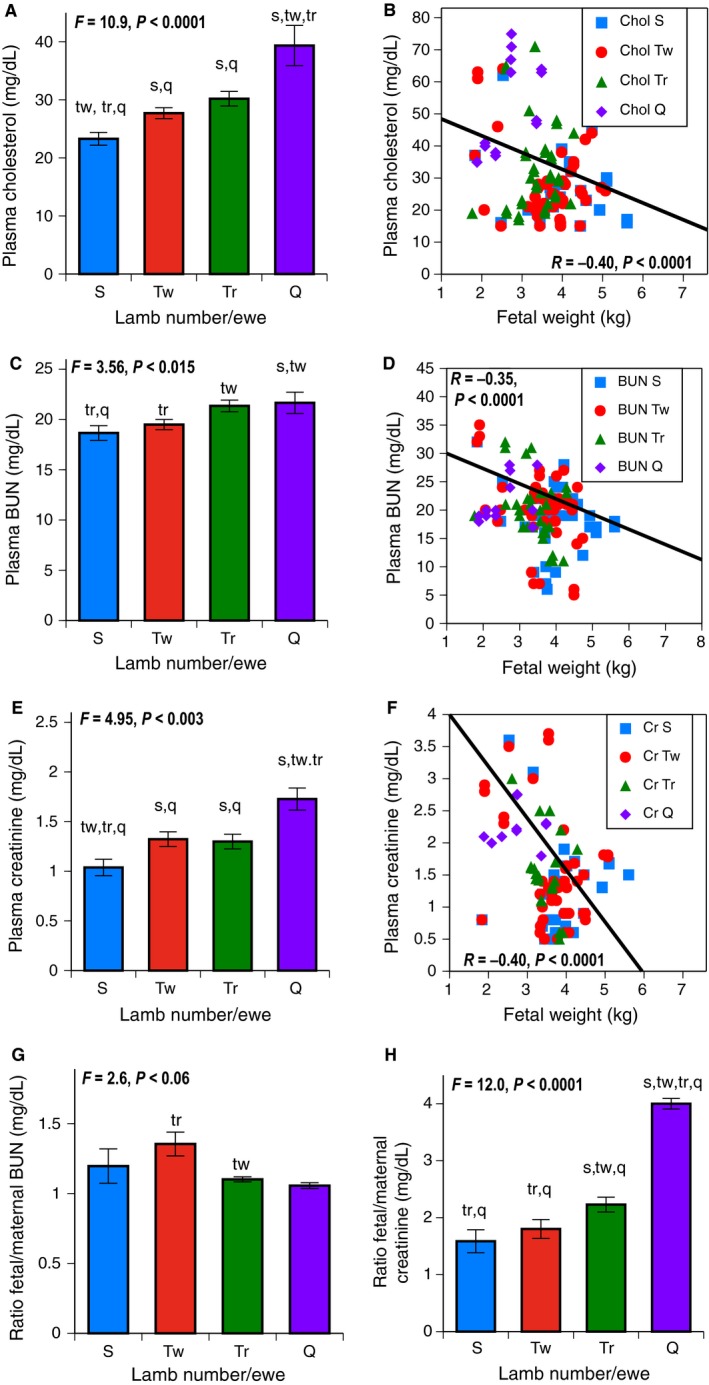
Placental and Fetal Excretory Function at GD130 on the vertical axis was examined based on fetal number per ewe with S (singletons in blue), Tw (twins in red), Tr (triplets in green), and Q (quadruplets/quintuplets in purple) shown on the horizontal axis. Error bars represent Means ± SEM. Letters on bar graphs indicate post hoc differences from other groups (s = differs from singletons, tw = differs from twin, tr = differs from triplet, q = differs from quad group). Regressions of growth parameters use the same color scheme, inset with S in squares, Tw in circles, Tr in triangles, and Q in diamonds. (A) Fetal Plasma Cholesterol levels (mg/dL); as fetal number per ewe increased, cholesterol rose, *P *< 0.0001. Sample sizes were 18 singletons, 58 twins, 42 triplets, and 8 quadruplets+. (B) Plasma Cholesterol Levels (mg/dL) and Fetal Weight (kg). Cholesterol was indirectly related to fetal weight, *P *< 0.0001. (C) Fetal Plasma Blood Urea Nitrogen (BUN) levels (mg/dL); as fetal number per ewe increased to triplets, BUN rose slightly, *P *< 0.015, with *post hoc* differences shown. Sample sizes were 18 singletons, 58 twins, 30 triplets, and 8 quadruplets+. (D) Fetal Plasma BUN levels (mg/dL) and fetal weight (kg). BUN was inversely related to fetal weight, *P *< 0.001. (E) Fetal Plasma Creatinine levels (mg/dL); as fetal number per ewe increased, creatinine rose, *P *< 0.0001. Sample sizes were 18 singletons, 58 twins, 30 triplets, and 8 quadruplets+. (F) Creatinine Levels (mg/dL) and Fetal Weight (mg/dL). Creatinine was inversely related to fetal weight, *P *< 0.0001. (G) Ratio of Fetal:Maternal Plasma BUN; although minimal change was appreciated by fetal number, (H) Ratio of Fetal:Maternal Plasma Creatinine rose, *P *< 0.0001.

Fetal plasma cholesterol levels are known to represent placental clearance from fetal production. At GD130, a stepwise increase in fetal plasma cholesterol was observed as fetal number per ewe rose (*P *<* *0.0001), (Fig. 4A). Fetal plasma cholesterol levels were indirectly related to fetal weight (*P *<* *0.0001), (Fig. 4B). Levels of fetal plasma BUN, recognized to be freely diffusible across the placenta (Robinson and Sprayberry 2009), were higher in triplets and quadruplets/quintuplets, compared to other groups (*P *<* *0.015), (Fig. 4C), and were inversely related to fetal weight (*P *<* *0.0001), (Fig. 4D). Fetal plasma creatinine rose with increasing fetal number per ewe, with twins and triplets similar (*P *<* *0.003), (Fig. 4E). Fetal plasma creatinine levels were inversely related to fetal weight (*P *<* *0.0001), (Fig. 4F). Ratio of fetal/maternal levels of both BUN and creatinine examined the relationship between maternal and fetal excretory function. The ratio of fetal/maternal BUN (Fig. 4G) did not differ by fetal number (*P *<* *0.06), but fetal/maternal plasma creatinine, although similar in singletons and twins, rose in a stepwise fashion in the triplet and quadruplet/quintuplet group (*P *<* *0.0001), (Fig. 4H).

### Demarcation as SGA status

We analyzed the data in dichotomous fashion (y/n) for body weight falling below 2.626 kg, SGA. All morphometric parameters were lower in the SGA group, including brain weight (g), *P *<* *0.015 for all, although brain weight (g) relative to body weight (kg) was still higher, *P *<* *0.005. Plasma levels of glucose, triglycerides, and LDH were lower, whereas creatinine and cholesterol were higher in the SGA group, *P *<* *0.015 for all. However, plasma levels of Alk Phosphatase, AST, and BUN did not differ when demarcated by SGA status.

## Discussion

This study defined normal plasma biochemical values for a group of nutritional measures, liver enzymes, and placental and fetal kidney excretory measures for late gestation fetal sheep, based on fetal number per ewe, showing stepwise space restriction inhibiting growth and disrupting placental nutrition, liver enzymes, and placental/fetal renal excretory tests. There is limited research on the effects of multifetal gestation on fetal wellbeing, but our study indicates that these fetuses exhibit short‐term metabolic consequences similar to other etiologies of IUGR. As anticipated, as fetal number rose, so did the percentage that weighed <10th percentile of our fetal singleton population. Qualitative comparisons (Table 1) from our current multifetal gestation ovine data are contrasted to findings on human fetuses with typical placental insufficiency‐induced IUGR; both have head sparing, with altered nutrition indices and increased accumulated wastes, but iron status was more protected in fetal sheep, compared to humans.

**Table 1:** Outline of placental efficiency parameters from Chem 20 and growth measurements into the categories of placental nutrition/growth, liver nutrition and function, iron nutrition, and waste excretion. Sources are listed below the table

Test Type	Test	Human fetuses limited space/IUGR	Sheep natural IUGR (multifetal gestation)
Placental nutrition/Growth	Kidney weight	↓	↓
	Liver weight	↓	↓
	Heart weight	↓	↓
	Brain weight	No change	No change
Liver nutrition & Function	AST	↑	No change
	GGT	↑	↓
	Glucose	↓	↓
	Alk Phos	Unknown	↓
	Triglycerides	↓	↓
Iron nutrition	Serum Iron	↓	No change
Waste excretion (Renal/Placental)	BUN	↑	↑
	Creatinine	↑	↑
	GFR	↓	↓
	Sodium (Electrolytes)	Unknown	No change
	Cholesterol	↑	↑

AST, Aspartamine transaminase; GGT, Gamma‐glutamyltranspeptidase; Alk Phos, Alkaline phosphatase; BUN, Blood urea nitrogen; GFR, Glomerular filtration rate.

Source: Cox et al. (1988); Meyer et al. (2010);Meyer‐Gesch et al. (2013); Sun et al. (2013); Verspyck et al. (1999); Alvino et al. (2008); Onyesom et al. (2009), Nieto‐Diaz et al. (1996); Rao and Georgieff (2007).

Arrows up or down denote values relatively to normally grown fetuses.

Loss in placentome number paralleled the observed drop in fetal and organ weights. The study found increased placentomal weight representing better placental efficiency, but similar to what we found in more extreme space restriction (Meyer et al. 2010; Meyer‐Gesch et al. 2013), after GD120, the ability to compensate in higher‐order multifetal gestation was limited. Thus, in this situation, placental efficiency does not accurately reflect functional indices including placental clearance or delivery of nutrients and metabolites. As anticipated, brain weights (g) did not fall, but rose when expressed as ratio of brain weight (g) per body weight, (kg) as fetal number per ewe rose, defining head‐sparing asymmetric growth restriction, with other organ weights proportionate to body weight. As anticipated, males weighed more than females. Brain weight fell slightly (86% of singletons) when fetal number reached four. Many of the fetal body weights fell below our flock's 10th percentile for singletons, representing growth restriction as a compensatory adaption (Barker 1992), potentially leading to developmental programming described by the Barker hypothesis (Fang 2005).

As fetal number rose, the stepwise lower fetal plasma glucose levels reflected ineffective placental facilitated diffusion because fetal liver contributes little to gluconeogenesis. The adaptive response to fetal hypoglycemia is to increase efficiency of tissue glucose uptake for fuel, as set forth by the thrifty phenotype hypothesis (Limesand et al. 2007). These findings are consistent with previous studies finding hypoglycemia in growth‐restricted fetal sheep (Morrison 2008), and with altered protein or mRNA expression of proteins within the insulin and gluconeogenic signaling pathways (Lie et al. 2014). The fall in glucose and triglyceride levels likely reflected lost substrate, as both are supplied by placenta. Finding lower circulating glucose and triglycerides as nutrient sources is consistent with that previously seen in multifetal gestation lambs after birth (Moallem et al. 2012). Current data are consistent with the finding of altered energy generation pathways in hearts and livers of twin fetuses and also to singleton lamb fetuses with preconception/preimplantation under nutrition that are epigenetically programmed in the anticipation of a future poor substrate supply (Lie et al. 2013, 2014).

In human fetal growth restriction, fetal plasma triglycerides, normally synthesized by placenta after the breakdown of maternal lipids, reflect the etiology, with abnormal placental implantation of preeclampsia raising triglycerides and malnutrition of placental insufficiency lowering triglycerides (Flores et al. 1974; Alvino et al. 2008). The current data support the later, with a threshold, nongraded drop in triglyceride levels in the quadruplet/quintuplet group. Low triglyceride levels might be explained by previous work that found epigenetic differences in genes involved in the adrenal IGF/growth hormone axis in late gestation twins, which may represent an adaptation to mitigate the poor placental substrate supply (Williams‐Wyss et al. 2014). Because poor placental triglyceride delivery can negatively impact cardiac growth and metabolism, increasing the risk of cardiovascular disease (Lie et al. 2013), the impact of multifetal gestation on future lipid metabolism should be further investigated.

Although fetal plasma levels of calcium and phosphorus did not differ, Alk Phosphatase, a vital hormone driving the mineral deposition in bone, fell in triplets and quadruplets groups. Alk Phosphatase, present in every tissue, rises with fetal maturation and rapid postnatal growth in premature humans (Tinnion and Embleton 2012). Placental–fetal Alk Phosphatase levels were previously found to parallel plasma glucose in IUGR (Onyesom et al. 2009). Maintaining fetal plasma calcium and phosphorus levels for fetal homeostatic enzymatic and metabolic pathways is vital, and thus lower Alk Phosphatase likely represents a diversion of minerals from bone development.

Liver enzymes were minimally changed, except that plasma AST levels were higher in the quadruplets/quintuplets. This finding could potentially reflect either liver ischemia or more likely intravascular hemolysis because ALT was not elevated. LDH, clinically considered a liver enzyme, was lower as fetal number per ewe increased, contrasting with a study showing total LDH was higher in human IUGR, although LDH 5 isoenzyme was reported lower in IUGR (Verspyck et al. 1999). LDH normally converts pyruvate, the final product of glycolysis, into lactate under hypoxia. Lactate accumulation is known to inhibit LDH synthesis and higher lactates were likely present because we, and others previously, found poorer oxygenation (Meyer et al. 2010; Meyer‐Gesch et al. 2013) or increased lactate levels (Morrison 2008) in growth‐restricted fetal sheep.

Limited data have been published on plasma electrolytes or fetal placental and renal excretory function parameters in multifetal gestation (Gibson and Lumbers 1999; Morrison 2008). Impaired placental excretion of wastes was supported by a graded rise in fetal plasma cholesterol level as fetal number rose (Wallach 1983). Fetal cholesterol and fetal adrenal glands both provide precursors to placental production of steroid hormones (Sanderson 2009). In contrast to our findings, cholesterol was previously reported to fall somewhat as fetal number rose in newborn lambs (Moallem et al. 2012), and it remains unclear why we found the opposite. The placenta serves to clear cholesterol from fetal production, so perhaps the additional 15 days to term gestation resulted in lower fetal cholesterol synthesis or neonatal factors dominated. The near‐complete fetal growth arrest in multifetal gestation became apparent between GD120 and GD130, and the additional days in gestation may further compromise transfer of fetally derived cholesterol to the placenta.

Greater levels of fetal creatinine and a higher ratio of fetal:maternal creatinine were likely due to poor excretory function. Work with fetal unilateral nephrectomy (halving fetal kidney function) reported a doubling of fetal plasma creatinine, concluding that excretory function is a joint function of placenta and fetal kidney (Anderson et al. 1999; Douglas‐Denton et al. 2002). In support, this study found 75% higher fetal creatinine with nephrons cut by 25% (Martin et al. 2010). Previously, lower plasma BUN and higher creatinine were seen in the smaller of discordant human twins (Arad et al. 2001), supporting that BUN reflected fetal protein, whereas creatinine reflected less placental–fetal renal excretion. If GFR fell by 50%, creatinine and potassium tests rose (Wallach 1983; Cheung and Lafayette 2013). In this study, the rise in plasma creatinine was greater than BUN, perhaps because BUN is more tightly regulated, or being of smaller molecular weight was more readily diffusible through the placenta (Meyer et al. 2010). Normally, initial fetal plasma creatinine levels reflect maternal levels, falling to infantile levels at 1 week, as renal blood doubles (Su and Stonestreet 2010). In human asymmetric IUGR, altered glomerular development and postnatal compensation predisposes to glomerular hyperfiltration and adult hypertension (Hinchliffe et al. 1992).

Strengths to the data include the large number of samples collected and the number that were growth restricted. Demarcating data as SGA or normally grown showed a similar direction for most biochemical values and supported that growth restriction strongly influenced multifetal gestation effects. A study limitation includes the slight delay in the time from anesthetic to fetal sampling, but finding that the mean maternal pO2 did not differ, the mean singleton fetal pO2 only mildly lower than reported values, and lack of fetal arteriovenous impact on measurements supported adequate fetal placental function despite this sampling delay.

This study quantitatively examines plasma biochemical parameters in multifetal gestation; providing normative data and aiding understanding of placental–fetal physiology. As fetal number rose, biochemical evidence supported increased functional adaptations in placental–fetal nutrition, placental and fetal renal excretory function, and fetal liver enzymes. Future research could further identify biomarkers and cut‐off limits to detect the most compromised fetal status (Sun et al. 2013) and to help understand mechanisms behind placental adaptation and developmental programming of long‐term effects. Understanding fetal blood biochemical indices in multifetal gestation may help understand lifelong health of multifetal gestation infants (Baschat 2004).

## Conflict of Interest

None declared.

## Supporting information




**Table S1.** Data at GD120 with 95% confidence intervals.
**Table S2.** Data at GD130 with 95% confidence intervals.

**Data S1.** Iron nutritional indices.
