# Supplementary material for: Increasing fetal ovine number per gestation alters fetal plasma clinical chemistry values
Source: Physiol Rep. 2016 Aug 26;4(16):e12905. doi: 10.14814/phy2.12905 (PMC5002913; doi:10.14814/phy2.12905)
Supplement: Supplementary file 1 — Table S1. Data at GD120 with 95% confidence intervals. Table S2. Data at GD130 with 95% confidence intervals. [file PHY2-4-e12905-s001.pdf]

SUPPLEMENTARY TABLE 1. Data at GD120 with 95% confidence intervals.

GD 120		n	Singletons	n	Twins	n	Triplets	n	Quadruplets +
Placental and Body Growth	Fetal Weight	15	2.23-6.34	48	2.08-3.5	54	1.99-3.34	16	1.27-2.73
	Kidney Weight	25	18.94-27.72	52	16.89-25.91	40	15.57-25.66	12	11.25-18.10
	Liver Weight	15	81.54-119.84	48	80.01-113.71	54	70.41-104.8	16	29.96-72.57
	Brain weight	15	32.69-41.65	48	30.55-44.07	52	35.6-43.41	16	28.1-35.91
Macronutrients and Minerals	Glucose	15	27.68-42.72	44	20.21-43.11	40	20.17-42.19	12	16.92-29.42
	Triglycer-ides	15	6.57-13.96	44	9.39-15.02	40	5.21-25.39	12	10.19-14.48
	Alk Phos	15	208.08-380.72	44	208.02-380.66	40	127.42-313.03	12	39.48-430.86
	Albumin	15	1.89-2.39	44	1.96-2.22	40	1.95-2.23	12	1.75-2
	Total Protein	15	3.11-3.77	44	3.14-3.92	40	3.21-3.62	12	2.93-3.19
Liver Enzymes	GGT	15	11.15-21.39	44	12.63-20.28	40	12.022-22.078	12	11.90-24.76
	AST	15	10.74-14.19	44	7.64-21.64	40	10.91-15.04	12	8.17-13.17
	ALT	15	2.41-5.86	44	2.78-6.04	40	3.39-5.41	11	5
	LDH	15	398.27-561.20	44	75.61-478.82	40	322.16-512.29	12	331.27-400.07
Excretory Function	Cholesterol	15	15.05-25.62	44	17.46-37.31	40	20.57-32.18	12	19.11-30.72
	Creatinine	13	0.67-1.19	40	.537-1.933	34	0.9-1.39	12	0.95-1.44
	BUN	15	17.26-22.08	44	13.30-23.92	40	16.56-27.44	12	14.21-27.12
	Sodium	15	138.97-144.90	44	138.23-145.19	40	138.15-143.86	12	139.27-143.9
Iron Nutrition	Serum Iron	15	171.10-269.57	44	217.09-282.78	40	179.47-273.68	12	134.93-226.41
	UIBC	4	16.6-131.2	17	30.3-135.8	14	21.5-86.7	0	
	TIBC	4	191.3-398.5	17	190.4-332.8	14	253-351.9	0	
	Tf saturation	4	66.8-88.2	17	42.0-90.3	14	74.9-91.4	0	
	Hb	12	9.49-17.01	20	10.78-15.88	34	9.48-14.27	8	11.38-12.67
	Hct	12	36.24-62.91	20	39.71-61.47	34	25.48-54.12	8	41.36-45.83

Abbreviations: IUGR = Intrauterine growth restriction, GD = Gestational day**,** AST = Aspartase transaminase, ALT = alanine transaminase, LDH = lactate dehydrogenase, GGT = gamma-glutamyltranspeptidase, Alk Phos = Alkaline phosphatase, BUN = Blood urea nitrogen, UIBC = unsaturated iron binding capacity, TIBC = total iron binding capacity, Tf saturation = transferrin saturation, Hb = Hemoglobin, Hct = hematocrit. Additional methods include plasma iron (μg/dL) by Cobas Integra, UIBC and TIBC (μg/dL), and Tf saturation (%) from Pointe Scientific, hemoglobin (Hb) (g/dL) and hematocrit (%) by pocH-100i, Sysmex

SUPPLEMENTARY TABLE 2. Data at GD130 with 95% confidence intervals.

GD 130		n	Singletons	n	Twins	n	Triplets	n	Quadruplets +
Placental and Body Growth	Fetal weight	14	3.45-6.19	45	2.35-4.95	34	2.78-3.91	8	1.81-3.02
	Kidney weight	18	23.40-36.98	58	19.52-28.26	42	17.82-25.88	14	14.73-21.02
	Liver weight	16	105.31-138.55	56	80.04-127.27	42	75.95-100.15	14	46.71-86.06
	Brain weight	16	4.64-51.87	52	37.67-48	40	39.16-53.16	14	37.19-41.48
Macronutrients and Minerals	Glucose	18	33.27-55.73	58	25.051-44.329	42	17.88-36.03	8	17.29-23.21
	Triglycer-ides	18	8.17-13.16	58	8.874-13.194	40	8.92-11.99	8	3.71-5.54
	Alk Phos	18	298.67-558.99	58	167.051-436.569	42	130.4-465.22	8	132.14-189.36
	Albumin	18	2.24-2.45	58	2.25-2.5	42	2.11-2.46	8	1.89-2.23
	Total Protein	16	3.66-3.95	56	3.53-4.04	42	3.32-4.09	14	3.30-4.16
Liver Enzymes	GGT	14	13.46-26.25	55	13.81-39.86	42	13.41-23.07	14	14.65-29.78
	AST	14	11-19.29	56	11.98-18.80	42	12.18-17.20	14	15.41-35.31
	ALT	16	3.47-5.53	56	3.25-5.53	36	3.89-5.28	14	2.29-6.57
	LDH	14	550.21-629.36	56	469.88-559.52	42	416.72-570.29	14	359.76-547.53
Excretory Function	Cholesterol	18	18.40-30.49	58	15.11-39.24	42	18.96-48.37	8	35.1-45.15
	Creatinine	18	0.64-1.734	58	0.527-2.287	30	0.99-2.33	8	1.87-2.13
	BUN	18	12.17-22.06	58	13.04-25.72	42	14.67-25.91	8	17.93-20.07
	Sodium	16	139.51-145.49	56	139.18-145.82	42	140.85-145.48	14	140.84-147.16
Iron Nutrition	Serum Iron	18	171.12-289.44	58	177.53-323.03	42	162.85-298.63		
	UIBC	6	32.54-121.94	17	30.25-135.83	14	21.45-86.73	0	
	TIBC	6	170.12-357.36	17	190.42-332.84	14	252.97-351.93	0	
	Tf saturation	6	58.19-84.01	17	42.04-90.28	14	74.85-91.37	0	
	Hb	16	10.96-14.60	37	11.39-14.07	42	11.16-15.43	14	11.08-17.88
	Hct	16	39.24-54.69	37	43.82-51.76	42	40.65-56.99	14	37.99-63.08

Abbreviations: IUGR = Intrauterine growth restriction, GD = Gestational day**,** AST = Aspartase transaminase, ALT = alanine transaminase, LDH = lactate dehydrogenase, GGT = gamma-glutamyltranspeptidase, Alk Phos = Alkaline phosphatase, BUN = Blood urea nitrogen, UIBC = unsaturated iron binding capacity, TIBC = total iron binding capacity, Tf saturation = transferrin saturation, Hb = Hemoglobin, Hct = hematocrit. Additional methods include plasma iron (µg/dL) by Cobas Integra, UIBC and TIBC (µg/dL), and Tf saturation (%) from Pointe Scientific, hemoglobin (Hb) (g/dL) and hematocrit (%) by pocH-100i, Sysmex.
