# Supplementary material for: Increasing fetal ovine number per gestation alters fetal plasma clinical chemistry values
Source: Physiol Rep. 2016 Aug 26;4(16):e12905. doi: 10.14814/phy2.12905 (PMC5002913; doi:10.14814/phy2.12905)
Supplement: Supplementary file 2 — Data S1. Iron nutritional indices. [file PHY2-4-e12905-s002.pdf]

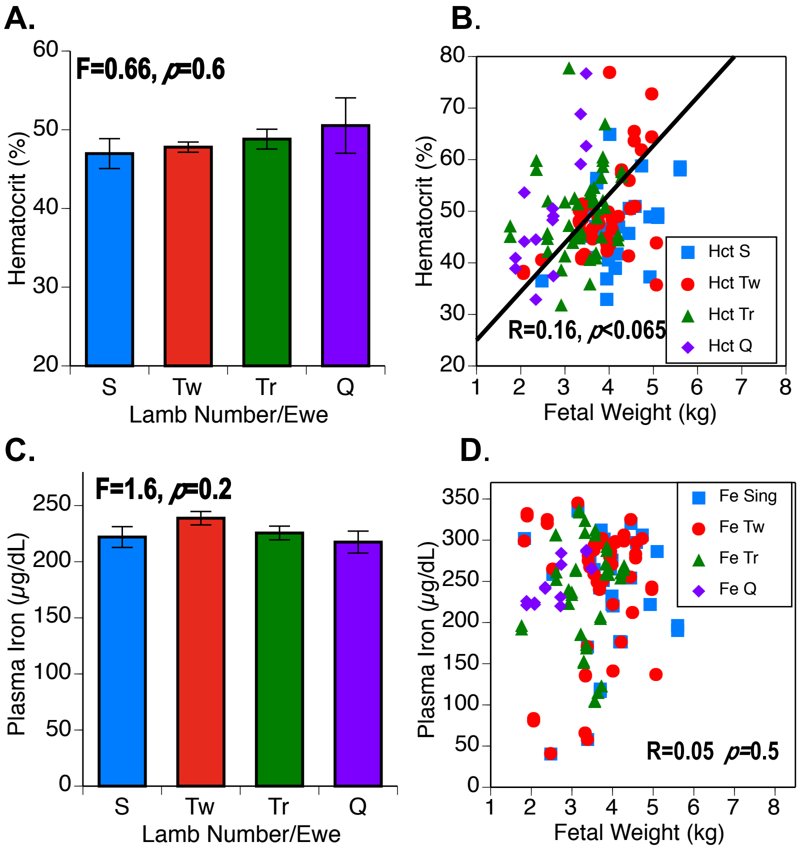

Supplementary Figure 1. Iron Nutritional on the vertical axis were examined based on fetal number per ewe with S (singletons blue), Tw (twins red), Tr (triplets green) and Q (quadruplets/quintuplets purple) shown on the horizontal axis. Error bars represent Means ± SEM. Lower case letters indicate *post hoc* differences (s from singletons, tw from twind, tr from tripletd, q from quadruplet/quintuplet). Regression lines follow the same color scheme, S in squares, Tw in circles, Tr in triangles, and Q in diamonds. **A. Fetal Hematocrit (%) and Fetal Weight (kg). B. Fetal Hematocrit (%) and Fetal Weight (kg);** Hematocrit (vertical axis) tended to be directly related to fetal weight, $p<0.065$. **C. Fetal Plasma Iron levels (mg/dL);** as fetal number per ewe increased, plasma iron was unchanged. **D. Fetal Plasma Iron Levels (µg/dL) and Fetal Weight (kg)**. Iron was not related to fetal weight.

Methods. Additional methods included plasma iron (µg/dL) (Cobas Integra) and hematocrit % (pocH-100i, Sysmex). **Results.** Although a subtle pattern of rise in hematocrit percentage was observed (***Fig. S1A)***, no significant relationship between hematocrit and fetal weight was evidenced, $p > 0.05$ (***Fig. S1B)***. No pattern emerged comparing fetal plasma iron per lamb number per ewe (***Fig. S1C)***, nor did any relationship between iron and fetal weight (***Fig. S1D)***. **Discussion**. In human IUGR liver iron is impaired, consistent with our previous description in fetal sheep with extreme uterine space restriction, including surgical restriction [Meyer KM, et al., Ovine surgical model of uterine space restriction: interactive effects of uterine anomalies and multifetal gestations on fetal and placental growth. Biol Reprod 2010;83(5):799-806]. In the current study, plasma iron levels were likely maintained via higher iron-binding capacity originating from fetal liver in the more moderate space restriction. During IUGR, the fetus compensates for hypoxemia by diverting iron away from liver, sustaining plasma iron level towards hemoglobin synthesis. However, in the current study, with less extreme natural increase in fetal number per ewe in this ruminant model, no trend was found in fetal plasma iron. In non-ruminants this may not be sustained and more work is necessary in other models.
